# An alternative route for the synthesis of silicon nanowires via porous anodic alumina masks

**DOI:** 10.1186/1556-276X-6-495

**Published:** 2011-08-17

**Authors:** Francisco Márquez, Carmen Morant, Vicente López, Félix Zamora, Teresa Campo, Eduardo Elizalde

**Affiliations:** 1 School of Science and Technology, University of Turabo, Gurabo, 00778 PR, USA; 2 Departamento de Física Aplicada C-XII, Universidad Autónoma de Madrid, Cantoblanco, 28049 Madrid, Spain; 3 Departamento de Química Inorgánica C-VIII, Universidad Autónoma de Madrid, Cantoblanco, 28049 Madrid, Spain

**Keywords:** Si NWs, AAO, masks, CVD

## Abstract

Amorphous Si nanowires have been directly synthesized by a thermal processing of Si substrates. This method involves the deposition of an anodic aluminum oxide mask on a crystalline Si (100) substrate. Fe, Au, and Pt thin films with thicknesses of ca. 30 nm deposited on the anodic aluminum oxide-Si substrates have been used as catalysts. During the thermal treatment of the samples, thin films of the metal catalysts are transformed in small nanoparticles incorporated within the pore structure of the anodic aluminum oxide mask, directly in contact with the Si substrate. These homogeneously distributed metal nanoparticles are responsible for the growth of Si nanowires with regular diameter by a simple heating process at 800°C in an Ar-H_2 _atmosphere and without an additional Si source. The synthesized Si nanowires have been characterized by field emission scanning electron microscopy, high-resolution transmission electron microscopy, X-ray photoelectron spectroscopy, and Raman.

## Introduction

One-dimensional semiconductor nanostructures have recently attracted intense research attention due to their novel physical properties [1-5], including electrical, magnetic, optical, and mechanical, and their potential for device applications in chemical and biological sensors, optoelectronic, transistors, etc. [6-8]. All these properties and potential applications can be modulated by controlling the chemical composition and the dimensionality of the nanowires, during the synthesis process [9]. Different methods have been used to synthesize Si nanowires (Si NWs) such as vapor-liquid-solid (VLS) process [10-12], laser ablation [13], chemical vapor deposition [14,15] or even thermal evaporation [16,17]. Electrodeposition techniques are an interesting alternative for nanowires growth due to the low cost and simplicity of the process [18-20]. This methodology uses a porous structure, which acts as a template, whose pores are electrochemically filled with the material of interest. This technique, however, has many technical problems to obtain nanowires with high aspect ratio.

In this study, we present an alternative procedure to those previously reported for the synthesis of nanowires. A porous structure (anodic aluminum oxide membrane) acts as an efficient template during the synthesis, controlling the dimensionality of the Si NWs. This methodology is based on the use of a porous membrane on which the catalyst is deposited. The use of silicon substrates as source for the Si NWs growth has recently been reported [21]. Nevertheless, in our study, the treatment temperature is clearly lower, the reaction time is reduced, the diameter of the Si NWs is regular and dependent on the synthesis parameters and the length of the nanowires is adjustable, controlling the growth time [22]. In this procedure, the diameter of the Si NWs can be related to the size of metal nanoparticles, whose dimensionality is adjustable by controlling the temperature, thickness of deposited material, and pore diameter of anodic alumina membrane used in the process [22]. In summary, it is noteworthy that the originality of this process lies in using the same substrate where the catalyst is deposited, as source of silicon, avoiding the use of complex systems with silicon-based vapor, together with a template that allow us to obtain silicon nanowires with regular dimensions.

## Experimental section

### Preparation of the anodic aluminum oxide templates

The synthesis of highly ordered porous alumina templates has been described elsewhere [23-28]. High-purity (99.999%) aluminum sheets, used as starting material, were degreased by using a mixture of HF, HNO_3_, HCl, and water (1:10:20:69,%*v*/*v*) and by ultrasonication in acetone. After that, the aluminum sheets were annealed under nitrogen atmosphere at 400°C for 3 h to remove mechanical stresses. Next, the aluminum foils were electropolished in a perchloric acid-ethanol solution (1:4, *v*/*v*) at 2°C. The anodization of the aluminum foils was made in two steps. The first anodization step was carried out using a constant voltage source (40 V) in a 0.3 M oxalic acid solution for 24 h and at a temperature around 1°C, then the oxide layer was removed by using a mixture of chromic and phosphoric acids at 30°C. The second anodization step was carried out for 3 h under identical conditions to the first anodization step. Afterwards, a saturated HgCl_2 _solution was used to dissolve the aluminum metal. Next, the barrier layer of the bottom part was removed and the pore diameter was widened by dipping the membrane in a 5 wt.% H_3_PO_4 _solution at 35°C for 20 min. The thickness of the free-standing porous alumina membrane was measured by field emission scanning electron microscopy (FESEM) to be 10 μm with a pore diameter of ca. 60 nm.

This anodic aluminum oxide (AAO) membrane was directly supported on a silicon (100) wafer. Other more compact Si substrates (Si (110) or Si (111)) are not able to generate any growth. The Si used in the growth process of nanowires is obtained from thermally generated defects on the surface of Si (100). These defects can be observed subsequently to the synthesis of Si NWs, as small cracks on the substrate, with loss of material. This Si is extracted from the single crystal and used in the growth of the Si NWs.

The adherence of the AAO template on the silicon substrate is produced by van der Waals forces and it can be substantially improved by wetting the AAO membrane in propan-2-ol/ethanol (2:1, *v*/*v*) mixture. After that, the template supported on the Si (100) was dried at 60°C overnight.

### Deposition of the catalyst on the AAO-Si sample

**Figure 1:**
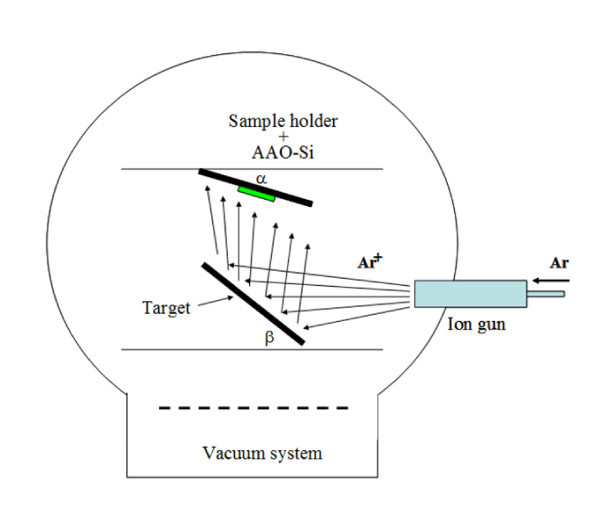
**Schematic representation of the single ion-beam sputtering system used for catalyst deposition on AAO-Si substrates**.

Different metals (30 nm) were deposited onto the AAO/Si samples by single ion-beam sputtering of a high-purity Au (99.999%, Goodfellow), Fe (99.95%, Goodfellow), and Pt (99.99%, Edelmetall) targets [24,29,30]. A referenced continuous Au, Fe, or Pt, film was simultaneously deposited on a Si (100) wafer to measure the thickness of the metal layer with a Taylor-Hobson Talystep profilometer. The experimental setup is shown in Figure 1.

**Figure 2:**
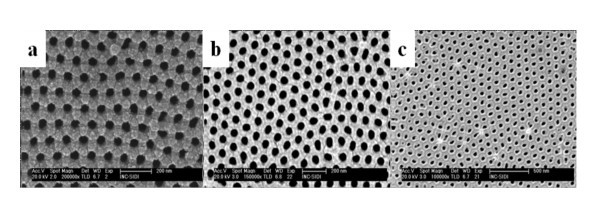
**FESEM images of the AAO-Si substrates after depositing a 30-nm-thick film**. A film of Fe (**a**), Au (**b**), and Pt (**c**) at room temperature.

During the metal deposition, the base vacuum was 10^-5 ^Pa and the argon pressure during sputtering was 0.1 Pa. In all cases, the deposition rate (measured with a quartz microbalance) was maintained at 2 nm min^-1^. During the sputtering, metal atoms are deposited on the AAO surface and also inside the inner pore surface. Figure 2 shows the FESEM image of AAO masks supported on Si (100) substrates after depositing a 30-nm-thick Fe (a), Au (b), and Pt (c) film at room temperature. As can be seen there, the metal deposition is homogeneously distributed due to the constant rotation of the sample holder that prevents the concentration of metal atoms in specific areas of the sample.

### Thermal treatment and growth of Si NWs

**Figure 3:**
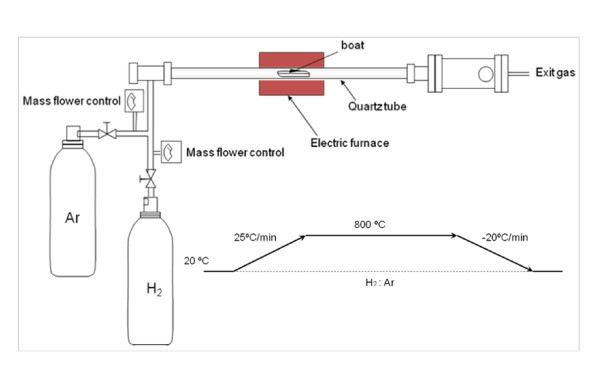
**Diagram of the CVD system and temperature ramps used in this study**.

The substrates were placed inside an alumina boat that was introduced in a tube furnace with a quartz reactor coupled, which was then heated at 800°C. The quartz reactor is coupled to a gas mixing system with mass flow controllers (see Figure 3).

Initially, 1,000 mL min^-1 ^of a mixture of hydrogen and argon (1:7 *v*/*v*) was flowed during the heating ramp (25°C min^-1^). When a temperature of 800°C was reached, samples were maintained in these conditions for 30 min. Finally, the flow of argon was readjusted to 1,000 mL min^-1 ^and hydrogen was stopped. After that, the cooling ramp was set at 20°C min^-1 ^under flowing argon during 5 h.

### Characterization methods

The morphology and microstructure of the Si NWs grown over AAO templates were analyzed by FESEM (Philips, FEG-XL30S, 20 kV, Philips Electronic Instruments Co., Chicago, IL, USA) and by high-resolution transmission electron microscopy (HRTEM, JEOL JEM-3000F, JEOL, Tokyo, Japan). Raman spectra were also recorded using a confocal Raman microscope (Renishaw RM2000, Renishaw plc, Wotton-under-Edge, UK) equipped with a laser source at 514 nm, a Leica microscope, and an electrically refrigerated CCD camera. The spectral resolution was set at 5 cm^-1^, laser power employed was less than 5 mW and the acquisition time was around 2 min.

HRTEM samples were prepared by dispersing the synthesized Si NWs in an ultrasound bath with ethanol followed by homogenization and placing 5 μL of this solution onto a copper grid coated with a lacy carbon film.

X-ray photoelectron spectroscopy (XPS) measurements were performed on a PHI 3027 system, by using the Mg Ka (1,253.6 eV) radiation of a twin anode in the constant analyzer energy mode with a pass energy of 50 eV.

## Results and discussion

### Morphological characterization

**Figure 4:**
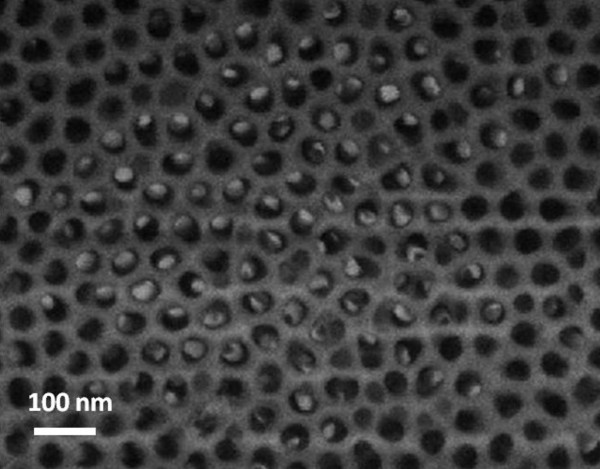
**FESEM image of the Pt catalyst incorporated by thermal effect within the pore structure**. Pore structure of the AAO mask-Si before the growth of nanowires.

During the initial stages of heat treatment, the catalyst deposited on the AAO-Si substrate melts and is incorporated within the porous alumina mask, resulting in nanoparticles with regular dimensions. These nanoparticles necessarily have a size smaller than the pores of the AAO mask and will be responsible for the constant dimensions of the synthesized nanowires. Figure 4 shows the surface of the AAO-Si substrate, once the molten catalyst has been incorporated within the porous structure of the membrane and before the treatment conditions allow the nanowires growth. As can be seen there, the catalyst can be observed as small particles inside the porous structure of the mask.

**Figure 5:**
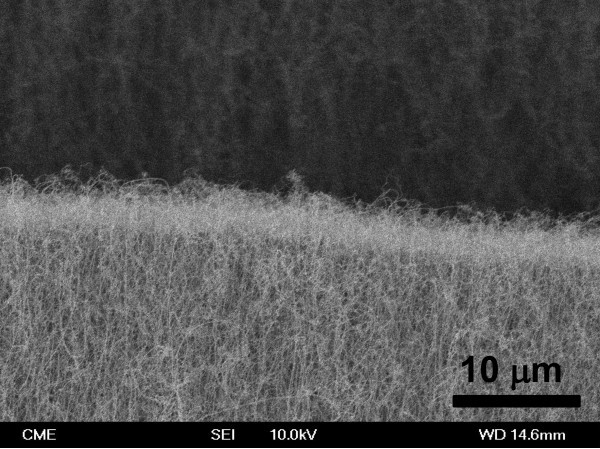
**FESEM image of the Si NWs obtained with Pt as catalyst**.

**Figure 6:**
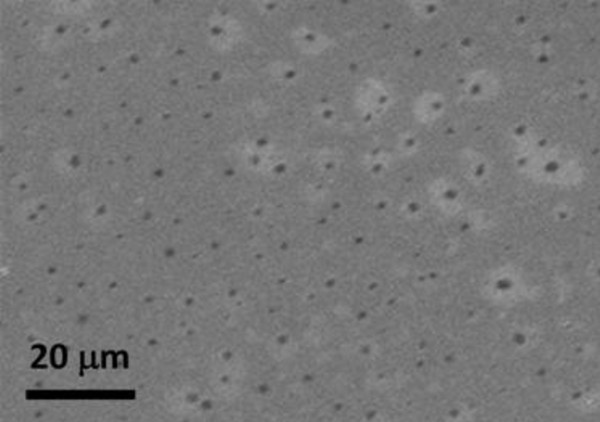
**SEM image of the Si (100) surface**. After growth, Si NWs and AAO template have been removed to reveal the dark points corresponding to defects and cracks generated on the susbstrate during the growth.

**Figure 7:**
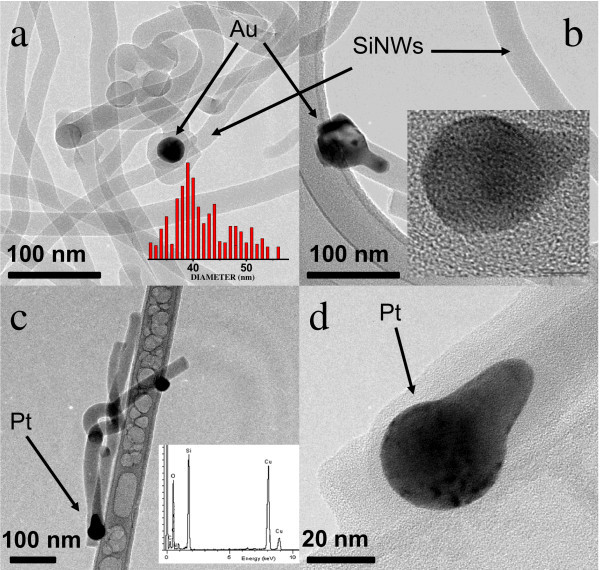
**HRTEM of Si NWs synthesized using Au (**a**, **b**) and Pt (**c**, **d**) as catalyst**. The inset of (a) shows a histogram of the Si NWs diameter distribution. The inset of (b) shows the Au nanoparticle. The inset of (c) corresponds to the EDX analysis of the Si NWs.

Figure 5 shows the FESEM image of the Si NWs obtained by using Pt as catalyst. Figure 5 shows a side view of the nanowires grown. As can be seen there, a high density of Si NWs emerges from the surface of the (AAO-Si) substrate. The use of Fe or Au catalysts produced similar growths although with a lower density of nanowires. Under these growth conditions, the only source of silicon is the substrate Si (100). We also tested other types of more compact silicon crystals, including silicon Si (111) or Si (011), but in these cases, there was no growth of nanowires. Possibly, this occurs because during the use of more compact substrates, the temperature used in treatment is not high enough to produce the evaporation of Si atoms. After the growth of nanowires, the Si (100) single crystal shows a large number of small cracks and holes on their surface. This silicon which has been removed from the crystal surface has been used in the synthesis of nanowires. Figure 6 shows a typical Si (100) surface obtained after thermal growth of Si NWs. As can be seen there, when the AAO mask and the Si NWs are removed from the substrate, the Si surface shows the presence of defects (dark points) with an average size and depth of around several micrometers. The morphology and size of the synthesized nanowires was also investigated by HRTEM. Figure 7 shows the HRTEM of Si NWs obtained by using Au (Fig. 7a and 7b) and Pt (Figure 7c, d) as catalysts, after dispersing by ultrasonic treatment of the nanowires in ethanol. It can be seen that several nanowires, with regular diameters are nucleated on catalyst nanoparticles. The metal nanoparticles are synthesized by using the AAO mask supported on the Si substrate as template. The thin metal layer deposited on the AAO-Si substrate is melted and incorporated inside the pores in contact with the Si surface. Since the nanoparticle size of the patterned catalyst is uniform, the grown nanowires are also uniform in diameter. The averaged pore size of the alumina mask, as determined by SEM, is about 60 nm. The lower nanoparticle size obtained from the alumina mask could be due to the sphericity induced by temperature, eventually generating particles of average size less than the predicted size. In all cases, the Si NWs are very long (tens of micrometers) with regular diameters of ca. 40 ± 10 nm. Inset of Figure 7a shows the histogram plot for the diameter distribution of the synthesized Si NWs.

Electron diffraction experiments on the Si NWs observed by TEM did not result in a diffraction pattern, evidencing the amorphous nature of this material. Upon closer inspection of the HRTEM images of the metal nanoparticles (inset of Figure 7b), it can be observed that the ordered fringes are demonstrating the crystalline nature of the metal particles generated during the melting process of the catalysts through the mask. On the other hand, EDXS measurements confirmed the composition of individual Si NWs to consist of silicon and oxygen (see the inset of Figure 7c). The oxygen signal is due to the presence of silicon oxides, possibly located on the surface.

### XPS characterization

**Figure 8:**
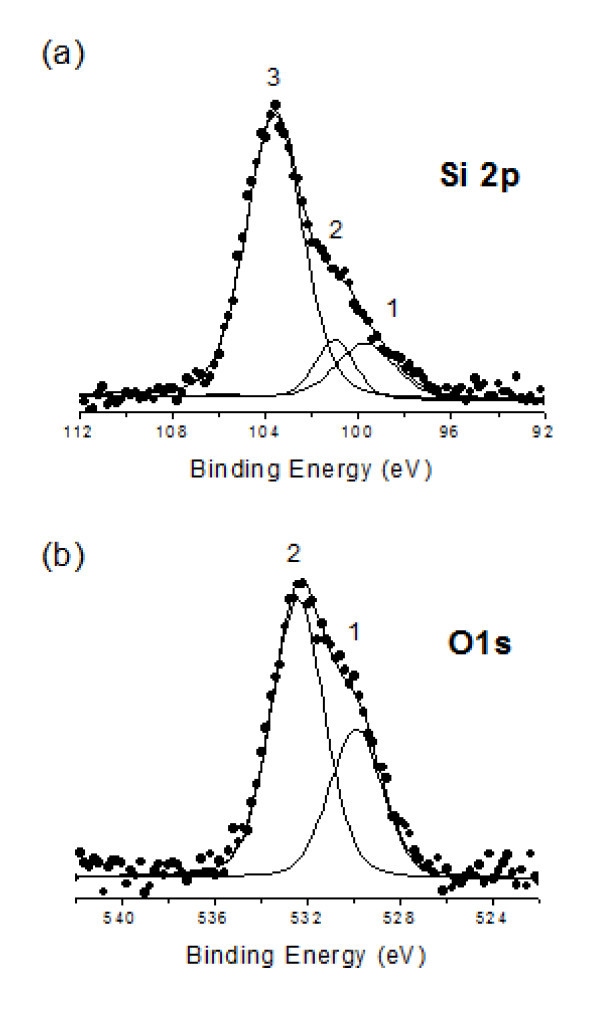
**XPS spectra of Si 2p (a) and O 1s (b), and the corresponding deconvolution analysis**.

Figure 8 shows the Si 2p and O 1s photoelectron spectra of Si NWs obtained by using Pt as catalyst. It is noteworthy that the XPS results obtained from nanowires grown using other catalysts (Fe or Au) show similar results. In order to eliminate the signal due to the Si substrate, XPS spectra were obtained after deposition of the Si NWs on a surface of highly oriented pyrolytic graphite (HOPG). The Si 2p spectrum (Figure 8a) shows a main peak and a shoulder at lower binding energies. The main peak at 103.6 eV (labeled as 3) has been attributed to Si in the oxidized form (SiO_2_) [31]. The shoulder at lower energy has been deconvoluted in two components at ca. 99.7 eV (labeled as 1) and at ca. 101 eV (labeled as 2). Interestingly, the peak 1 has been attributed to Si^0 ^[31]. The peak 2, required for the deconvolution, can be ascribed to the presence of substoichiometric Si oxides (SiO_*x*_) [31]. Figure 8b shows the XPS spectrum of O 1s. As can be seen there, this band is not symmetric and it has been deconvolved in two components. The main peak observed at 532.4 eV (labeled as 2) has been attributed to oxygen in SiO_2 _[31]. In a similar way as was observed with the Si 2p spectrum, the peak at 529.9 eV (labeled as 1) has been assigned to the presence of substoichiometric oxides (SiO_x_) [31] and possibly to oxygen adsorbed on the HOPG substrate.

The results obtained by XPS and EDX indicate that the Si NWs are constituted by Si^0^, SiO_2_, and substoichiometric silicon oxides (SiO_*x*_). Moreover, studies of electron diffraction by TEM reveal that the Si NWs are amorphous in nature. Possibly, Si NWs are composed of a Si^0 ^core surrounded by a silicon oxide shell. Different studies on the synthesis of amorphous silica nanowires consider that the explanation for the amorphous nanowires production is the growth temperature. In fact, when temperature is not high enough, recrystallization is not produced and, in our case, we have used a constant growth temperature of 800°C.

### Raman characterization

**Figure 9:**
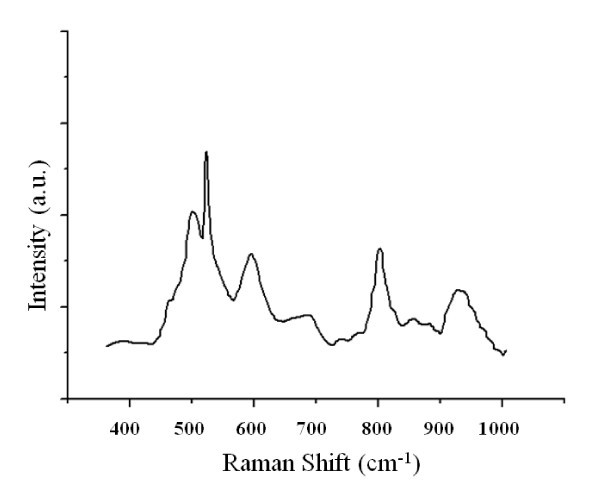
**Raman spectra of Si NWs**.

Figure 9 shows the Raman spectrum of the Si NWs grown by using Pt as catalyst. As can be seen there, a sharp Raman line at ca. 512 cm^-1 ^is observed. This peak can be related to the Si-Si stretching mode. Nevertheless, Raman peaks at more than 510 cm^-1 ^(typically around 520 cm^-1^) have been justified as due to crystalline silicon. The above studies reveal that there was no trace of a crystalline phase in the synthesized Si NWs. On the other hand, XPS analysis indicates the presence of silicon suboxides and in this way, the Raman shift at positions near to that corresponding to crystalline phases can be attributed to the effect of the oxygen deficiency [32].

The peak at ca. 485 cm^-1 ^(m) can be justified as due to the bond Si-O of amorphous SiO_2 _or also to substoichiometric oxides. The Raman peak at ca. 584 cm^-1 ^(m) has been assigned to Si-O-Si bending of silicon oxides. The broad peak at 931 cm^-1 ^is due to the stretching mode of amorphous Si-Si (vibration that is also observed at 512 cm^-1^). Finally, the Figure 9 shows three peaks at ca. 678 (w), 798 (m), and 860 cm^-1 ^(w), that have been associated to the stretching mode of Si-O.

## Conclusions

In the present work, we have used AAO masks to synthesize Si NWs on Si (100) substrates, by using Fe, Au, and Pt as catalysts. In this approach, the Si (100) substrate acted as both silicon source and growth substrate, allowing the synthesis of Si NWs with regular dimensions.

The growth mechanism corresponds to a VLS process. In this mechanism, the growth happens when silicon from the Si (100) substrate diffuses into the alloy puddle, favoring the melting of Si into the alloy [33].

The diameter of the nanowires ranged from ca. 30-50 nm, with an average size of ca. 40 nm and was related to the pore size of the AAO mask. HRTEM revealed the amorphous nature of the Si NWs, possibly due to the low growth temperature used during the synthesis. EDX, XPS, and Raman have shown that they are composed of Si^0 ^and silicon oxides (SiO_2_-SiO_*x*_) possibly forming a Si^0 ^core surrounded by a silicon oxide shell. Nevertheless, further research is needed to clarify this point.

## Competing interests

The authors declare that they have no competing interests.

## Authors' contributions

FM, CM, VL, FZ, TC, and EE synthesized different samples. FM, CM, TC, and EE characterized the synthesized samples by Raman, XPS, SEM, and TEM.

## References

[B1] Adu KW Gutierrez HR Kim UJ Sumanasekera GU Ecklund PC Confined phonons in Si nanowires Nano Lett 2005 5 409 414 10.1021/nl0486259 15755085

[B2] Akiyama T Nakamura K Ito T Structures and electronic properties of Si nanowires grown along the [1 1 0] direction: role of surface reconstruction Surf Sci 2008 602 3033 3337 10.1016/j.susc.2008.08.002

[B3] Clément N Tonneau D Dallaporta H Bouchiat V Fraboulet D Mariole D Gautier J Safarov V Electronic transport properties of single-crystal silicon nanowires fabricated using an atomic force microscope Phys E Low-dimens Systems and Nanostruct 2002 13 999 1002 10.1016/S1386-9477(02)00288-6

[B4] Dalchiele EA Martín F Leinen D Marotti RE Ramos-Barrado JR Synthesis, structure and photoelectrochemical properties of single crystalline silicon nanowire arrays Thin Solid Films 2009 518 1804 1808

[B5] Guo CS Yang XB Zhang RQ Remarkable effects of surface dihydride configurations in electronic properties of < 110 > silicon nanowires Solid State Commun 2009 149 1666 1669 10.1016/j.ssc.2009.06.022

[B6] Bi X Agarwal A Yang KL Oligopeptide-modified silicon nanowire arrays as multichannel metal ion sensors Biosens Bioelectron 2009 24 3248 2351 10.1016/j.bios.2009.04.007 19443202

[B7] Bi X Wong WL Ji W Agarwal A Balasubramanian N Yang KL Development of electrochemical calcium sensors by using silicon nanowires modified with phosphotyrosine Biosens Bioelectron 2008 23 1442 1448 10.1016/j.bios.2007.12.012 18242974

[B8] Gao C Deng SR Wan J Lu BR Liu R Huq E Qu XP Chen Y 22 nm silicon nanowire gas sensor fabricated by trilayer nanoimprint and wet etching Microelectron Engineer 2010 87 927 930 10.1016/j.mee.2009.11.173

[B9] An X Meng GW Wei Q Kong M Zang L SiO_2 _Nanowires Growing on Hexagonally Arranged Circular Patterns Surrounded by TiO_2_ Phys Chem B 2006 110 222 226 10.1021/jp055463b 16471525

[B10] David T Buttard D Hertog MD Gentile P Baron T Ferret P Rouvière JL Silicon nanowires grown in nanoporous alumina matrices on < 100 > oriented silicon substrates investigated by electron microscopy Superlatt Microstruct 2008 44 354 361 10.1016/j.spmi.2007.10.011

[B11] Bae J Kulkarni NN Zhou JP Ekerdt JG Shih CK VLS growth of Si nanocones using Ga and Al catalysts J Cryst Growth 2008 310 4407 4411 10.1016/j.jcrysgro.2008.06.084

[B12] Zhang J Xu B Yang Y Jiang F Li J Wang X Wang S Catalyzed-assisted growth of well-aligned silicon oxide nanowires J Non-Cryst Solids 2006 352 2859 2862 10.1016/j.jnoncrysol.2006.02.088

[B13] Fukata N Oshima T Okada N Kizuka T Tsurui T Ito S Murakami K Phonon confinement in silicon nanowires synthesized by laser ablation Phys B: Condensed Matter 2006 376-377 864 867

[B14] Lu M Li MK Kong LB Guo XY Li HL Silicon quantum-wires arrays synthesized by chemical vapor deposition and its micro-structural properties Chem Phys Lett 2003 374 542 547 10.1016/S0009-2614(03)00747-4

[B15] Liu ZQ Zhou WY Sun LF Tang DS Zou XP Li YB Wang CY Wang G Xie SS Growth of amorphous silicon nanowires Chem Phys Lett 2001 341 523 528 10.1016/S0009-2614(01)00513-9

[B16] Chen J Pan Y Wu R Growth mechanism of twinned SiC nanowires synthesized by a simple thermal evaporation method Phys E: Low-dimensional Systems and Nanostructures 2010 42 2335 2340 10.1016/j.physe.2010.05.016

[B17] Zhang RQ Chu TS Cheung HF Wang N Lee ST Mechanism of oxide-assisted nucleation and growth of silicon nanostructures Mater Sci Engineer C 2001 16 31 35 10.1016/S0928-4931(01)00295-8

[B18] Kim K Kim M Cho SM Pulsed electrodeposition of palladium nanowire arrays using AAO template Mater Chem Phys 2006 96 278 282 10.1016/j.matchemphys.2005.07.013

[B19] Peppler K Janek J Template assisted solid state electrochemical growth of silver micro and nanowires Electrochim Acta 2007 53 319 323 10.1016/j.electacta.2006.12.054

[B20] Xu CL Li H Zhao GY Li HL Electrodeposition and magnetic properties of Ni nanowire arrays on anodic aluminum oxide/Ti/Si substrate Appl Surf Sci 2006 253 1399 1403 10.1016/j.apsusc.2006.02.056

[B21] Park HK Yang B Kim SW Kim GH Young DH Kim S H Maeng SL Formation of silicon oxide nanowires directly from Au/Si and Pd-Au/Si substrates Phys E: Low-dimensional Systems and Nanostructures 2007 37 158 162 10.1016/j.physe.2006.08.003

[B22] Márquez F Morant C Elizalde E Zamora F López V Synthesis of silicon nanowires 2010 Spanish Patent P201030501 10.1186/1556-276X-6-495 PMC3212010 21849077

[B23] Yanagishita T Nishio K Masuda H Fabrication of metal nanohole arrays with high aspect ratios using two-step replication of anodic porous alumina Adv Mater 2005 17 2241 2243 10.1002/adma.200500249

[B24] Márquez F Morant F Pirota KR Borrás A Sanz JM Elizalde E Fabrication of ordered crystalline zirconium nanoporous membranes by an one-step procedure Nano Today 2009 4 21 26 10.1016/j.nantod.2008.10.012

[B25] Navas D Hernández-Vélez M Asenjo A Jaafar M Baldonedo JL Vázquez M Preparation and magnetic characterization of Ni membranes with controlled highly ordered nanohole arrays IEEE Trans Magn 2006 42 3057 3059

[B26] Masuda H Fukuda K Ordered metal nanohole arrays made by a two-step replication of honeycomb structures of anodic alumina Science 1995 268 1466 1468 10.1126/science.268.5216.1466 17843666

[B27] Li A Müller F Birner A Nielsch K Gösele U Fabrication and micro-structuring of hexagonally ordered two-dimensional nanopore arrays in anodic alumina Adv Mater 1999 11 483 487 10.1002/(SICI)1521-4095(199904)11:6<483::AID-ADMA483>3.0.CO;2-I

[B28] Lei Y Chim WK Zhang Z Zhou T Zhang L Meng G Phillipp F Ordered nanoporous nickel films and their magnetic properties Chem Phys Lett 2003 380 313 318 10.1016/j.cplett.2003.09.025

[B29] Morant C Márquez F Campo T Sanz JM Elizalde E Niobium and hafnium grown on porous membranes Thin Solid Films 2010 518 6799 6803 10.1016/j.tsf.2010.06.034

[B30] Márquez F Morant C Campo T Sanz JM Elizalde E Ordered Metal Nanotube Arrays Fabricated by PVD J Nanosci Nanotechnol 2010 10 1115 1119 10.1166/jnn.2010.1841 20352765

[B31] Moulder JF Stickle NF Sobol PE Bomben KD Chastain J, King RC Handbook of X-ray Photoelectron Spectroscopy 1995 Eden Prairie: Physical Electronics

[B32] Nishikawa H Shiryawa T Nakamura R Ohki Y Nagaswa K Hama Y Photoluminescence from defect centers in high-purity silica glasses observed under 7.9-eV excitation Physical Review B 1992 45 586 591 10.1103/PhysRevB.45.586 10001096

[B33] Paulose M Varghese OK Grimes CA Synthesis of gold-silica composite nanowires through solid-liquid-solid phase growth J Nanosci Nanotechnol 2003 3 341 346 10.1166/jnn.2003.209 14598450

